# COVID-19 in Children with Congenital Heart Diseases: A Multicenter Case Series from Iran

**DOI:** 10.1155/2021/6690695

**Published:** 2021-04-17

**Authors:** Hassan Esmaeeli, Mehdi Ghaderian, Keyhan Sayadpour Zanjani, Seyyedeh Fatemeh Ghalibafan, Mehrzad Mahdizadeh, Mohammad Hassan Aelami

**Affiliations:** ^1^Cardiology Department, School of Medicine, Gorgan University of Medical Sciences, Gorgan, Iran; ^2^Pediatric Cardiovascular Research Center, Cardiovascular Research Institute, Isfahan University of Medical Sciences, Isfahan, Iran; ^3^Cardiology Division, Pediatric Department, Children's Medical Center (Pediatric Center of Excellence), Tehran University of Medical Sciences, Tehran, Iran; ^4^Mashhad University of Medical Sciences, Mashhad, Iran; ^5^Radiology Division, Children's Medical Center (Pediatric Center of Excellence), Tehran University of Medical Sciences, Tehran, Iran; ^6^Department of Pediatrics and Hand Hygiene and Infection Control Research Center, Imam Reza Hospital, Mashhad University of Medical Sciences, Mashhad, Iran

## Abstract

**Background:**

Promptly discovering and counteracting COVID-19 is critical as it could have catastrophic effects. As an asymptomatic group, children are highly susceptible to be misdiagnosed, especially those suffering from underlying diseases. Furthermore, discriminating the direct effects of the virus from those of the underlying diseases can pose a dilemma to physicians. This case series aims to determine the relationship between COVID-19 and various types of congenial heart disease among children. *Patients and Methods*. Seven patients from three different medical centers were enrolled. Their detailed demographic information, past medical history, symptoms, type of congenital heart diseases, imaging tests, laboratory tests, medications, and outcomes were analyzed.

**Results:**

The patients included 4 infants, 1 child, and 2 adolescents, with a median age of 9 months and a majority of boys. All of them had either obstructive lesions (right or left ventricular outflow tracts) or significant pulmonary hypertension. The more common clinical symptoms were cough, dyspnea, and fever. Two patients did not survive the illness.

**Conclusion:**

Prompt treatment of patients with a combination of COVID-19 and severe obstructive cardiac pathology or pulmonary hypertension is essential due to a risk for serious and/or fatal consequences.

## 1. Introduction

In December 2019, the SARS-CoV-2 infection (COVID-19) was initially discovered in China as a new emerging disease. Subsequently, it has spread rapidly among other countries including Iran, and eventually, it was declared by the World Health Organization as a “pandemic” on 11^th^ March 2020. It is also reported that the prevalence of symptoms is lower in children compared to adults [1]. Coronary artery diseases, heart failure, and cardiac arrhythmia have been associated with increased mortality among adults with COVID-19 [2].

There is still a lack of knowledge and studies regarding children and adolescents suffering from congenital heart diseases (CHD) and COVID-19. SARS-CoV-2 infection may have direct cardiac involvement or indirect effects on the heart through respiratory and other organ involvements. Theoretically, the effects of this infection on a pressure- or volume-overloaded heart in the setting of CHD may be more serious. In addition, risk factors for the severity of COVID-19 in patients with CHD are unknown. Genetic syndromes are usually seen in patients with CHD, but their effects in this situation are unknown. Discovering the impacts of CHD on the course and outcome of COVID-19 enables physicians to set priorities for emergency interventions. In this case series, we reported seven pediatric CHD patients from three medical centers in Iran.

## 2. Case Presentation

The patients' data were collected after an online webinar where Iranian pediatric cardiologists shared experiences about their COVID-19 patients. Hospital databases and responsible physicians were the sources of data. No written consent has been obtained from the patients as there are no patient identifiable data included in this case series.

  Inclusion criteria were as follows: (1) positive reverse transcriptase polymerase chain reaction (RT-PCR) assay for coronavirus RNA from nasopharyngeal swab specimens, (2) symptoms attributable to COVID-19, (3) presence of CHD, and (4) age ≤18 years.
  Exclusion criteria were as follows: (1) mild obstructive lesions, (2) small defects with negligible shunt or hemodynamic disturbance, (3) insignificant cardiac diseases such as bicuspid aortic valve or mitral valve prolapse, (4) positive PCR test without any symptoms in a patient with CHD, and (5) multisystem inflammatory syndrome in children, SARS-CoV-2 myocarditis, and similar diseases.

**Figure 1:**
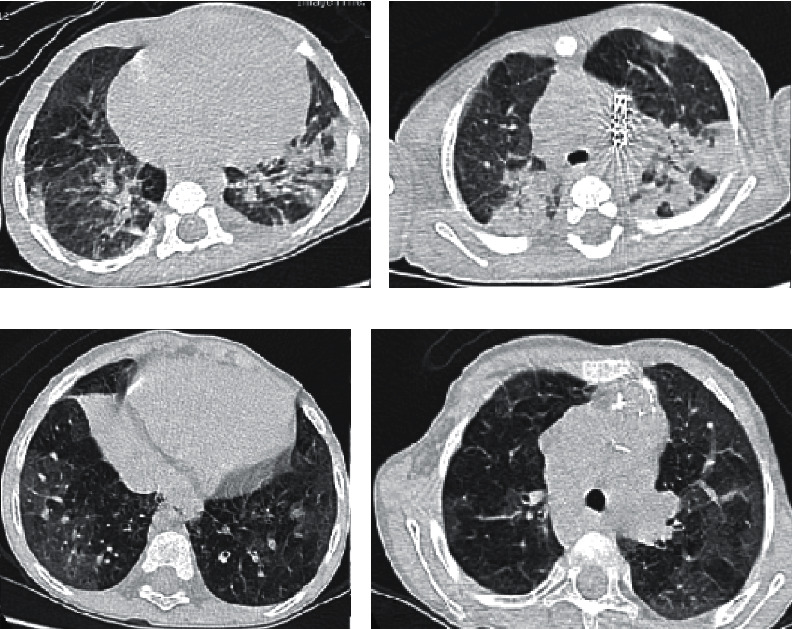
CT scan (a, b) of patient 1 with subpleural alveolar consolidations, deformed interlobular septal thickening, and mosaic attenuation. Ductal stent can be seen in (b). CT scan (c, d) of patient 2 with diffuse ground-glass opacities.

**Figure 2:**
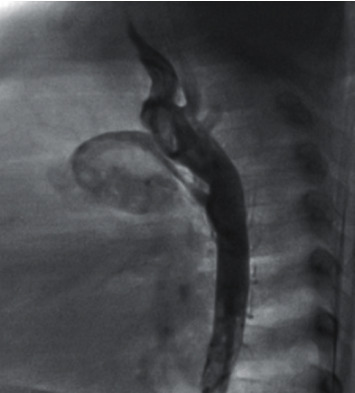
Lateral angiogram showing severe neointimal proliferation inside the stent, especially at its pulmonary end.

The primary data are summarized in Table 1. The patients included 4 infants, 1 child, and 2 adolescents with a median age of 9 months (range 2 months to 14 years). The majority of them were male (71%). Four patients had obstructive lesions: three had lesions in the right ventricular outflow tract (RVOT) and one had lesions in the left ventricular outflow tract (LVOT). Two patients had Eisenmenger complex, and one had significant pulmonary hypertension (PH). Cough was the most common symptom, followed by fever, dyspnea, cyanosis, restlessness, poor feeding, and edema. Laboratory data are presented in Table 2. Highly elevated erythrocyte sedimentation rate (ESR) or C-reactive protein (CRP) was seen in four patients. One patient had mild lymphopenia. Thrombocytopenia was seen in three patients during the hospitalization. Decreased oxygen saturation was seen in 5 patients. Treatments and outcome data are summarized in Table 3. The median hospital stay was 11 days (range 4 to 28 days), and the median ICU stay was 3 days (range 0 to 15 days). The mortality rate was 29% (Figures 1 and 2).

  Patient 1: he had borderline hypoplastic left heart syndrome (HLHS) and underwent balloon aortic valvuloplasty as a neonate. Consequently, the degree of aortic stenosis was reduced with negligible aortic insufficiency. He remained prostaglandin-dependent, and hence ductal stenting was performed using a 5 × 24 PALMAZ Blue stent (Cordis, USA). While awaiting bilateral pulmonary artery banding (PAB), interval increase in the size of the left ventricle encouraged us to proceed with a biventricular approach. A serious viral respiratory infection caused increased cyanosis and suprasystemic pulmonary artery pressure at the age of 5 months. Eisenmenger complex was confirmed during a diagnostic catheterization. Since he had typical mild symptoms of COVID-19, he was admitted to the hospital. The second swab test was positive for SARS-CoV-2. CT scan showed lung parenchymal involvement (Figures 1(a) and 1(b)). His condition suddenly deteriorated with increasing edema and elevated CRP. Worsening of RV function was documented by echocardiography. While attempting to start drugs against a probable cytokine storm (corticosteroids), the patient died of bradycardia and cardiac arrest.
  Patient 2: the patient was referred for peripheral edema. At the age of 2.5 months, he underwent surgical correction of truncus arteriosus using a 14 mm homograft at our hospital. Then, he had been followed at another institution with moderate homograft stenosis with a peak pressure gradient (PG) of 40 mmHg at the last follow-up. Initial echocardiogram at our hospital showed severe homograft stenosis (65 mmHg peak PG) in the presence of right ventricular (RV) failure, tricuspid valve regurgitation (peak PG 80 mmHg), and PH (PI with 45 mmHg peak PG). As he came from a highly epidemic area, a SARS-CoV-2 PCR test was performed which was negative. Although homograft stenosis could be seen, the cause of PH and RV failure was not clear. A CT scan showed severe lung disease (Figures 1(c) and 1(d)). The second SARS-CoV-2 PCR was positive. Follow-up echocardiograms after discharge showed persistence of RV failure in spite of two negative PCR tests. Because of his suboptimal lung condition for a homograft exchange operation, we decided to relieve RVOT stenosis by stenting. Cardiac catheterization demonstrated homograft stenosis (32 mmHg peak PG) and near systemic peak RV pressure (56 mmHg versus 61 mmHg aortic peak systolic pressure). Mixed venous sample had 67% oxygen saturation, and the calculated pulmonary vascular resistance and the ratio of pulmonary to systemic vascular resistances were 2 and 0.37 Woods unit, respectively. A PALMAZ Genesis XD stent 2910 (Cordis, USA) on a 14 × 30 millimeter Cristal balloon (Balt, France) was deployed across the stenotic homograft which resulted in a decrease in peak PG to 17 mmHg.
  After 2 months, RV failure and pulmonary symptoms persisted. Therefore, we suspected additional reasons for his condition. We found two comorbidities that may account for his lung condition: severe gastroesophageal reflux and severe T-cell immune deficiency. Fluorescence in situ hybridization for 22q11 deletion was negative. However, other forms of DiGeorge syndrome were highly suspected. Aggressive antireflux drugs and prophylactic antibiotics were administered.
  Patient 3: she was 14 years old, awaiting aortic valve repair or replacement at the time of presentation. She presented with respiratory distress and decreasing oxygen saturation that led to intubation, mechanical ventilation, decreased consciousness, and finally death after three days of hospitalization. Her condition did not permit us to perform a CT scan.
  Patient 4: a 4-month-old male infant had fever of unknown origin for 15 days. High ESR with a history of being in a highly pandemic region raised the suspicion of COVID-19. The first PCR was positive. He was retreated in a noncritical care setting and eventually discharged in fair condition.
  Patient 5: a 2-month-old male infant was our youngest patient. He had a typical presentation of COVID-19 with cough, fever, restlessness and poor feeding, and a large ventricular septal defect (VSD). His first PCR test was positive. He stayed only in the noncritical setting and was discharged in an acceptable condition.
  Patient 6: he was an adolescent with Down syndrome, unoperated complete atrioventricular septal defect, and Eisenmenger complex. He demonstrated typical manifestations of COVID-19 and was discharged after 4 days in the intensive care unit and 2 days in the COVID-19 ward.
  Patient 7: a 4.5-month-old female infant with pulmonary atresia and intact ventricular septum with a history of patent ductus arteriosus (PDA) stenting in the neonatal period was referred to us for increasing cyanosis. Oxygen saturation had decreased from 80% at the previous visit to 65%, although the stent was patent. The parents had symptoms (cough in the father and severe headache in the mother). PCR testing for SARS-CoV-2 was positive in all three. The patient was admitted and was discharged after 4 weeks. The main reason for this prolonged stay was persistent cyanosis even after improvement of the laboratory and other clinical findings. For this reason, we did a diagnostic angiography one month later. Aortic angiogram (Figure 2) discovered severe neointimal proliferation inside the stent, mainly at its pulmonary end. The patient underwent surgical pulmonary valvotomy and PDA ligation.

## 3. Discussion

In this case series as described, COVID-19 presented with significant symptomatology in pediatric patients with underlying CHD. Although children with COVID-19 are often asymptomatic, special underlying congenital cardiac diseases can predispose to serious and rarely fatal outcomes. Of particular importance is the burden of disease in patients with severe untreated obstructive lesions (RVOT or LVOT) and patients with significant PH. Intervention to correct the severe obstructive lesions or PH can be recommended especially in epidemic areas. In the cohort described by Sabatino et al., there were more COVID-19 patients with an underlying cardiac diagnosis of VSD, pulmonary atresia, and transposition of the great vessels [3].

The clinical symptoms, CT scan findings, and laboratory parameters of COVID-19 are similar among children with normal heart or CHD [1]. We observed increased degree of cyanosis in patients with underlying cyanotic congenital heart disease. Heart failure can be seen due to myocarditis or as a manifestation of multisystem inflammatory syndrome in children [4]. In two patients (patients 2 and 7), we observed mild transient ventricular dysfunction without any rise in cardiac enzymes. The right ventricle was dominant (HLHS after PDA stenting with RV failure) in one and the left in the other (pulmonary atresia and intact ventricular septum after PDA stenting). Thromboembolic events can be part of COVID-19 [5]. In patient 7 with a PDA stent, we found severe neointimal proliferation that led to increasing and persistent cyanosis. This finding is, however, comparatively rare among our cohort of patients with PDA stents.

The relationship between angiotensin-converting enzyme (ACE) inhibitors or angiotensin receptor blocker (ARB) and COVID-19 is still controversial. The SARS-CoV-2 binds to the SARS-coronavirus receptor ACE2, and there are concerns about susceptibility of the users to the infection due to increased receptor expression [2]. Two of our patients were receiving captopril before the infection. CT scan as a valuable imaging modality can determine the severity of COVID-19 pneumonia and also confirm the diagnosis [6]. Differentiating changes from underlying cardiac disease to those from the infection can be difficult. CT scans of the lung performed in 6 patients were consistent with SARS-CoV-2 infection.

With two mortalities, COVID-19 was relatively severe in our patients. Providing instructions for proper hand hygiene in caregivers and healthcare workers, right use of masks, and social distancing are crucial to prevent this infection [7].

## 4. Conclusion

Patients with ventricular pressure overload in the form of severe stenosis of the RVOT or LVOT, and/or significant PH/Eisenmenger complex may be at a higher risk for symptomatic or severe COVID-19. Prompt treatment of these diseases especially in epidemic areas is recommended.

## Figures and Tables

**Table 1:** Demographic data, symptoms, CT scan, echocardiographic data, and treatment history of the patients.

Patients	Age	Sex	CHD	Symptoms	CT scan	Echo	Medication	Intervention	Surgery
1	9M	M	HLHS, Eisenmenger complex	Cough, low-grade fever, restlessness, increased cyanosis	Subpleural alveolar consolidation, deformed interlobular septal thickening, mosaic attenuation	RV failure	Sildenafil, captopril, aspirin, digoxin	BAV PDA stenting	—
2	5Y	M	Truncus arteriosus, homograft stenosis	Edema, dyspnea, cyanosis	Diffuse GGO	RV failure	Sildenafil, captopril	—	Surgical repair by homograft
3	14Y	F	Severe AS, moderate AI	Cough, dyspnea, low-grade fever	Alveolar consolidation mostly in the periphery of the left lower lobe	—	—	—	On the operation waiting list
4	4M	M	Moderate PS	Cough, fever	Mosaic attenuation, air trapping linear atelectasis	—	—	—	—
5	2M	M	VSD, PH	Cough, fever, restlessness, poor feeding	GGO, air trapping, mosaic pattern	—	—	—	—
6	14Y	M	CAVSD, Eisenmenger complex	Cough dyspnea	Subpleural patchy consolidation, mild pleural effusion	—	Bosentan, furosemide	—	—
7	4.5M	F	PA, IVS	Increased cyanosis, dyspnea, restlessness	Peribronchial thickening, mosaic attenuation	LV failure	Aspirin	PDA stenting	—

CHD: congenital heart disease; echo: echocardiography; M: male; HLHS: hypoplastic left heart syndrome; RV: right ventricle; BAV: bicuspid aortic valve; PDA: patent ductus arteriosus; F: female; GGO: ground-glass opacities; AS: aortic stenosis; AI: aortic insufficiency; PS: pulmonary stenosis; PA: pulmonary atresia; PH: pulmonary hypertension; IVS: intact ventricular septum; LV: left ventricle.

**Table 2:** Laboratory data of the patients at presentation.

Patient	PCR	ESR	CRP	Lymphocytes	Platelets	Saturation^∗^*∗*
1	+	1	230	1830	76	60 (75)
2	+	1	16	2400	372	58
3	+	32	++	1050	89	89
4	+	65	Weakly +	8300	207	95
5	+	7	Negative	3700	389	92
6	+	6	17	1450	132	70 (80)
7	+	4	54	4500	226	65 (80)

ESR: erythrocyte sedimentation rate; CRP: C-reactive protein. *∗*^∗^If a patient was cyanotic before the infection, saturation before the infection was written in parentheses.

**Table 3:** Treatments and outcomes.

Patient	Hospital stay	ICU stay	Ventilation	Outcome
1	17	15	Mechanical	Death
2	11	8	Spontaneous	Discharge
3	4	3	Mechanical	Death
4	12	0	Spontaneous	Discharge
5	11	0	Spontaneous	Discharge
6	9	7	Spontaneous	Discharge
7	28	3	Spontaneous	Discharge
